# Selected Metabolites of Biofunctional Importance from Edible Fruits of Forest Shrubs

**DOI:** 10.3390/molecules30010073

**Published:** 2024-12-28

**Authors:** Anna Przybylska-Balcerek, Kinga Stuper-Szablewska

**Affiliations:** Department of Chemistry, Poznań University of Life Sciences, 60-637 Poznan, Poland; kinga.stuper@up.poznan.pl

**Keywords:** secondary metabolites, polyphenols, phenolic acids, flavonoids, natural pigments, carotenoids, chlorophyll, anthocyanins

## Abstract

This study focused on determining the content of bioactive compounds in selected fruits of wild shrubs. The plants selected for the study were from the Rosaceae and Adoxaceae families. Particular attention should be paid to the fruits of plants commonly growing in Poland (temperate climate), such as *Crataegus monogyna*, *Sorbus aucuparia*, *Viburnum opulus,* and *Sambucus nigra*. The study aimed to deepen the knowledge of the content of selected secondary metabolites, such as phenolic acids, flavonoids, flavonoid glycosides, and their antioxidant properties, as well as natural dyes. During this study, chromatographic and spectrophotometric methods were used to determine the quantitative profile of the above-mentioned secondary metabolites of wild plant fruits. The quantitative profile of 16 phenolic acids, 9 flavonoids, 5 organic acids, 13 flavonoid glycosides, and 3 natural dyes was determined. Based on the studies, it was noted that the qualitative and quantitative profile of the bioactive compounds differs not only depending on the species but also on the location where the plant grows. A statistical analysis showed significant differences (*p* < 0.05) in the content of phenols and flavonoids in fruits collected from different locations. Interestingly, differences were also observed within the species, probably depending on the geographical location and composition of the soil in which the plants were grown.

## 1. Introduction

**Figure 1:**
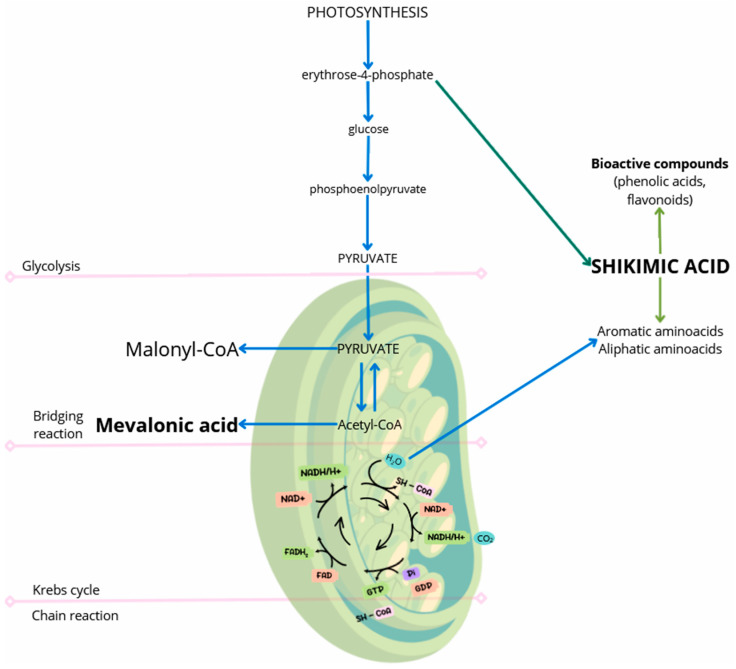
Metabolism of compounds in a plant cell [5].

**Figure 2:**
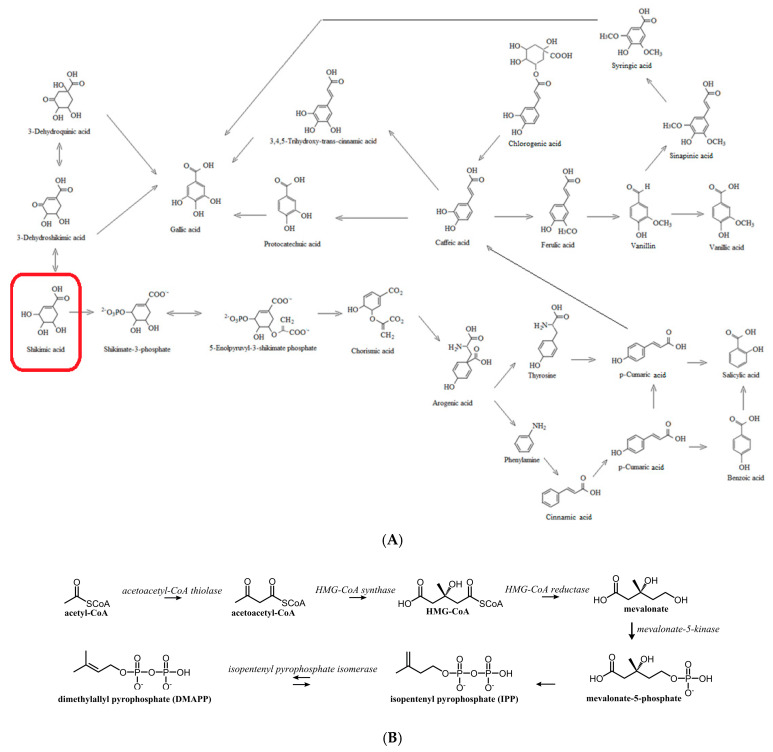
(**A**) Shikimic acid metabolism (own study, based on the literature) [6,7,8]. (**B**) Mevalonate pathway [9].

The plant can be considered a storehouse of a huge number of chemical substances biosynthesized in many metabolic pathways, including photosynthesis, glycolysis, the Krebs cycle, and the shikimic acid pathway. Metabolites are a heterogeneous group of natural metabolic products with low molecular weight and diverse structure and biological activity [1]. The intensive development of analytical methods, extraction techniques, and isolation methods has not only allowed for the discovery of new, biologically active chemical substances but also helped in the understanding of their structures and mechanisms of action. Metabolites are produced at the cellular level, as a result of the biochemical reactions of all living organisms. During the transformations, various types of intermediate metabolites and end products are formed. The term metabolites was quite general. Therefore, Albrecht Kossel, in 1891 [2], introduced the division into secondary metabolites (derived from primary metabolites under the influence of various environmental stresses, such as light, temperature, and various metals, using several metabolic pathways; these compounds do not directly affect the survival of the organism but only the functioning of a given organism) and primary metabolites (intermediate products of the Krebs cycle; compounds necessary for the growth and reproduction of the organism, such as amino acids, nucleic acids, carbohydrates, and fats). It is now known that it is difficult to draw a rigid boundary between primary and secondary metabolism, and the balance and dynamics between them depend on the plant’s growth and the intensity of environmental stressors [3,4]. Secondary metabolites play a key role in the communication of the plant with the environment, perform adaptive functions (alkaloids, cyanogenic glycosides, polyphenols, terpenoids, etc.), and are unique to individual families, species, and even plant organs. The ability of different taxa to biosynthesize specific secondary metabolites is reflected in the term “specific substances” or “secondary metabolites”. Functional levels of plant metabolism are closely interconnected by the fact that the starting substances for the synthesis of secondary metabolites are compounds derived from primary metabolism (Figure 1 and Figure 2).

Metabolism consists of two directions of transformations: catabolism and anabolism. Active metabolites are formed as a result of anabolic transformations. Polyphenol synthesis takes place via two main metabolic pathways: the shikimic and the acetomalonic pathways. In the case of the first, the main precursor is shikimic acid, from which aromatic amino acids (L-phenylalanine, L-tyrosine, and L-tryptophan) are biosynthesized, as well as polyphenols (Figure 2A,B). Their substrates are products of primary metabolism: phosphoenolpyruvate from glycolysis and erythro-4-phosphate from the pentose phosphate pathway. As a result of the action of plant enzymes usually activated by stress factors, shikimic acid can give rise to various hydroxybenzoic acids. In contrast, during the deamination of L-phenylalanine, the enzyme phenylalanine ammonia-lyase (PAL) produces trans-cinnamic acid. This acid is considered to be one of the most important phenolic metabolites in plant cells, being a bridge between the metabolism of aromatic amino acids and polyphenols. The acetomalonate pathway in higher plants is usually coupled with the phenylpropanoid pathway and leads to the formation of flavonoids. The most important bioactive substances include flavonoid glycosides, flavonoids, phenolic acids, organic acids, and others. In human nutrition, secondary metabolites have a variety of effects on the body, including improving the functioning of the digestive system, affecting the correct body weight, regulating the lipid profile of the blood, and reducing the risk of circulatory system diseases and cancers [6].

The results of many studies indicate that the best sources of bioactive compounds are the fruits of wild plants, as exemplified by those from the Rosaceae Juss and Adoxaceae families. The largest number of genera occur in the temperate climate zone (Central Europe, North America, and Asia). These are plant families of great economic importance. They include many wild and cultivated fruit trees and shrubs, as well as ornamental and medicinal plants. Commonly found in Polish forests and thickets are, among others, *Crataegus monogyna* Jacq (*C. monogyna*)., *Sorbus aucuparia* L. (*S. aucuparia*) from the *Rosaceae Juss*. family and *Viburnum opulus* L. (*Opulus vulgaris* Borkh., *V. opulus*) and *Sambucus nigra* L. (*S. nigra*) from the *Adoxaceae* family.

Although the health-promoting properties of the fruits of the above plants have been well known and appreciated for many years, research is still being conducted to determine the detailed chemical composition of these fruits, especially the content of polyphenolic compounds and organic acids and their beneficial effects on the body that result from their antioxidant activity. The available literature data do not exhaust the topic of the content of bioactive substances and antioxidant activity of the above fruits. The aim of the study was to determine the qualitative and quantitative profile of selected secondary metabolites in the fruits of wild *C. monogyna* and *S. aucuparia* from the *Rosaceae Juss* family and *V. opulus* and *S. nigra* from the *Adoxaceae* family.

## 2. Results and Discussion

### 2.1. Antioxidant Activity, the Total Phenolic Content, Total Phenolic Acid Content, and Total Flavonoid Content

Samples of selected fruits of forest shrubs (common *C. monogyna*., common *S. aucuparia* from the *Rosaceae* Juss. family, and *V. opulus* and *S. nigra* from the *Adoxaceae* family), were collected from two locations in Poland. The method of extract preparation was optimized for the specificity and chemical composition of the material. The main subject of the study was the secondary metabolites, mainly bioactive compounds with antioxidant properties. The mean values of the results are presented in Table 1 below. The first stage of the study was the analysis of antioxidant activity measured using the ABTS^+^ method (ABTS^+^ μmol Trolox equivalents/kg—antioxidant activity applying an improved 2,2-azino-bis-3-ethylbenzothiazoline-6-sulfonic acid). High values of antioxidant activity were measured during the analyses of the fruits of *C. monogyna*, *V. opulus,* and *S. nigra,* indicating the presence of antioxidant compounds. The highest mean value was determined for *C. monogyna* (2537–2844 μmol Trolox equivalents/kg dry matter (d.m.)), and it was twice as high as in the other samples tested. Similar studies were conducted by Froehlicher et al. [6], who also examined the antioxidant activity of *C. monogyna* fruits from France. Their results show that fruit extracts have a lower antioxidant activity compared to extracts from other morphological parts of this plant. In the study [10] by Keser et al., it was observed that there is a correlation between ABTS^+^ and the content of polyphenols. Tadic et al. [11] reported that *C. monogyna* fruits from Serbia also showed ABTS^+^ activity. Apak et al. [12] reported that the mechanism of the ABTS^+^ reaction is still unclear, depending on individual antioxidants as well as reaction conditions. The present study also describes the antioxidant activity in rowanberries. Based on the conducted studies, it was found that the antioxidant activity of ABTS^+^ was 1204–1244 μmol Trolox equivalents/kg d.m. fruit, depending on the location. Similar studies were conducted by Sarv et al. [13], during which they noticed that *S. aucuparia* fruits of the Likernaja, Burka, Rubinovaja, and Granatnaja varieties had above-average values of antiradical capacity (666 and 1068 μmol Trolox equivalents/kg d.m.). In turn, Jurikova et al. [14] and Kampuss et al. [15] based on their studies detected the highest antioxidant activity of the Likernaja variety, which is found among other hybrids. The present studies on the antioxidant activity of ABTS+ were also conducted on two other fruits of wild plants belonging to the *Adoxaceae* family (*V. opulus*, *S.nigra*). In previous studies, Przybylska-Balcerek et al. also analyzed the antioxidant activity, measured by ABTS^+^, in *S. nigra* fruits from 36 different locations. At that time, high antioxidant activity values were found, ranging from 647.21 to 721.25 μmol Trolox equivalents/kg d.m. fruit [16].

Then, during the tests, the total polyphenol content was measured (TPC). Based on the conducted studies, the highest TPC was found in the fruits of *S. nigra* (4177–5042 mg gallic acid equivalents (GAE)/100 g of fruit d.m.) and *C. monogyna* (4299–4322 mg GAE/100 g of fruit d.m). In the fruits of *Sorbus aucuparia* L., the TPC content was two times lower, and in the fruits of *V. opulus,* it was four times lower. The varied TPC values among the tested samples prove that the fruits of wild plants can be a valuable source of polyphenols for food and pharmaceutical purposes [17,18]. For comparison, according to the literature, TPC in 100 g of *S. nigra* fruit is variable and amounts to e.g., 25.87–38.87 mg/g of d.m. [19], 827 mg/g of d.m. [20], 1336 mg/g of d.m. [21], or 513.6 mg/g of d.m. [22]. Sarv et al. [13] in their study showed that the TPC values for 16 sweet *S. aucuparia* varieties ranged from 2.53 to 15.05 mg GAE/g dry weight, from 0.53 to 14.8 mg GAE/g dry weight, and from 15.97 to 44.68 mg GAE/g dry weight for whole fruits. The highest levels were found for the Likernaja, Burka, Rubinovaja, and Granatnaja varieties. The Likernaja and Burka varieties are rowan–chokeberry hybrids, *S. aucuparia* × Aronia melanocarpa, and *Sorbus aria* × Aronia arbutifolia, respectively, while Rubinovaja is × Sorbopyrus (*S. aucuparia* × Pyrus), and Granatnaja is × Sorbocrataegus (*S. aucuparia* × Crataegus). The TPCs in the fruits of Likernaja and Burka [13] cultivars were 15.05 and 14.78 mg GAE/g d.m., respectively. These results are similar to the TPC values reported by Kampuss et al. [15], who showed that the highest TPC values were in Likernaja (484.9 mg/100 d.m.), among eight other *S. aucuparia* cultivars. Hukkanen et al. [23] tested many *S. aucuparia* cultivars and found the highest TPC values for Rubinovaja and Burka, 1014 and 820 mg/100 g d.m. of fruits, respectively. In the study of Hukkanen et al. [23], the Burka variety was characterized by the highest content of anthocyanins among sweet *S. aucuparia* fruits. Polka et al. [24] in their studies showed that the TPC content in *V. opulus* fruits was in the range of 3.51–3.98 g/100 g d.m. and significantly exceeded the concentration of carotenoids. For comparison, the content of phenolic compounds was estimated at 0.68–0.83 g/100 g in fruits grown in the Czech Republic and 0.40–0.73 g/100 g in *Viburnum opulus* fruits from Russia [24]. The total content of phenolic compounds in fruits grown in Turkey or Lithuania was 0.62–0.99 g/100 g of d.m. and 0.75–1.46 g/100 g of d.m., respectively [25,26,27].

The next stage of the study was the measurement of the total content of free phenolic acids (TAC) and total flavonoid content (TFC). The highest content of flavonoid TFC was found in the fruits of *C. monogyna* (1041–1057 mg RUTE/100 g of fruit d.m.), while the fruits of *S. nigra* and *S. aucuparia* contained two times less. The results obtained for TAC and TFC are presented in Table 1 Accordingly, the fruits of *S. nigra* and *C. monogyna* have the highest average value of TAC and TFC compared to the average value of the other fruits. For comparison, the TFCs in the fruits of *Viburnum opulus*, as reported in the literature, ranged from 0.20 to 0.49 g (Polka et al., 2019) [24]. At that time, flavonoids constituted 56.5% of all phenolic compounds in the fruits of *Viburnum opulus*. During their research, Goudjil et al. [28] determined the TFC in *C. monogyna* fruit extracts (TFC: 151.38 ± 1.05 mg QE/g D.M.). Moreover, both in the present study and the study in Goudjil et al. [28], the TFC results in *C. monogyna* fruits are significantly higher compared to those obtained from the fruits collected in Konya, Turkey (TFC: 36.91 ± 0.17 mg QE/g) [29]. Moreover, the TFC in the fruits of *V. opulus* was also determined by Çolak et al. [30] during their studies. According to them, the TFC ranged from 1463 to 3163 mg quercetin L^−1^. Meanwhile, Ersoy et al. [31] showed that TFC accounted for 27.3–37.4% of the TPC in fresh fruits of *V. opulus*.

The results of the study suggest that these wild fruits are a significant source of polyphenols that may have potential applications in food and pharmaceuticals.

### 2.2. Natural Pigments

In the next stage, the content of natural dyes in fruit extracts was analyzed. These dyes determine the color of ripe fruit. The analysis showed the presence of carotenoids, chlorophylls, and anthocyanins (Table 2). Based on the conducted research, it was noted that carotenoids dominated in the fruits of *C. monogyna* (206.3–211.5 mg/kg d.m.). It was similar in the case of *S. aucuparia* (134.2–151.1 mg/kg d.m.) and *V. opulus* (51.8–56.3 mg/kg d.m.). It was different in the case of *S. nigra* fruit because the compounds with the highest content were anthocyanins (517.4–579.6 mg/kg d.m.). The literature also contains information on the content of carotenoids in the tested fruits. Aurori et al. [32] found 95.68 ± 0.297 µg/g d.m. for the total concentration of carotenoids in the fruits of *S. aucuparia*. In terms of the total content of carotenoids, the results were similar to those of the Latvian varieties [15]. Carotenoids were found in higher concentrations in the Moldavian varieties of *S. aucuparia* and lower concentrations in the Serbian and Montenegrin varieties [33,34]. In turn, the content of total carotenoids in the studies by Kajszczak et al. [35] was 2.70 mg/100 g (d.m).

The data included in reference publications indicate that the total content of anthocyanins in 100 g of fresh *S. nigra* fruit varied significantly and amounted to 200–1000 mg [36], 465.1 mg [22], 272.87 mg [37], 863.89 mg [38], and 1265 mg [39]. The differences in the content of anthocyanins could be caused by differences between *S. nigra* varieties and weather conditions during ripening. According to data from scientific publications, the content of anthocyanins in 100 g of fresh fruit was 529–664 mg in the Haschberg variety, 877 and 1815 mg in the Sampo variety, and 846 and 1634 mg in the Samyl variety.

The results of the study suggest that these wild fruits are a significant source of natural pigments that may have potential applications in food and pharmaceuticals.

### 2.3. Phenolic Acids, Flavonoids, Organic Acids, and Flavonoid Glycosides

Based on the conducted research, the quantitative profile of 25 bioactive compounds in the above-mentioned fruits of wild plants was determined. These compounds were selected as a common denominator of the qualitative profile of phenolic compounds. Therefore, 16 phenolic acids (gallic acid, 4-hydroxybenzoic acid, 2,5-dihydroxobenzoic acid, vanilic acid, caffeic acid, syringic acid, coumaric acid, *p-*cumaric acid, benzoic acid, ferulic acid, sinapic acid, *t-*cinnamic acid, chlorogenic acid, protocatechuic acid, rozmaric acid, and salicylic acid) and 8 flavonoids (apigenin, catechin, kaempferol, luteolin, naringenin, quercetin, rutin, and vitexin) 1 phenolic aldehyde (vanilin) were analyzed (Table 3). Based on the conducted studies, it was noted that both the qualitative and quantitative profiles of the bioactive compounds differ depending on the fruit variety. *C. monogyna* fruits are characterized by a high content of chlorogenic acid (1158–1199 mg/kg), which is twice as high as in *V. opulus* fruits and three times higher than in *S. nigra* fruits. In turn, the most characteristic flavonoid for *C. monogyna* fruits is quercetin (1057–1102 mg/kg). Half the content of quercetin was found in *S. nigra* fruits. Analyzing the profile of bioactive compounds in *S. aucuparia* fruits, the highest content of naringenin was found (610–613 mg/kg), while in *S. nigra* fruits, this flavonoid was 10 times lower. A characteristic feature of *S. nigra* fruits is the high content of rutin (1798–2044 mg/kg), as well as the identification of phenolic compounds that were not found in the other fruits tested. These are gallic acid, 4-hydroxybenzoic acid, 2,5-dihydroxobenzoic acid, vanilic acid, caffeic acid, syringic acid, coumaric acid, vanilin acid, *p-*cumaric acid, benzoic acid, ferulic acid, sinapic acid, rozmaric acid, salicylic acid, apigenin, katechin, kempferol, and luteolin. Both during this and previous studies, the authors determined the profile of bioactive compounds in *S. nigra* fruits [16]. Based on the obtained results, it was found that the dominant metabolites were rutin, quercetin, and chlorogenic acid. It was noted that, among the tested fruits of wild plants, only in *S. nigra* were 23 out of 25 analyzed phenolic compounds detected. An analysis allowing for the identification of individual phenolic acids was also used by Sarv et al. in their studies, who found the presence of chlorogenic and neochlorogenic acids, ranging from 1.07 to 4.59 mg/g dry weight and from 0.75 to 6.13 mg/g dry weight, respectively [13]. The highest contents of chlorogenic acid were determined in fruit and juice samples of Sahharnaja, Bussinka, Angri, and wild *S. aucuparia* varieties. Neochlorogenic acids, followed by chlorogenic acids, were the most dominant phenolic acids. These results were similar to the results of the study by Bobinaitė et al. [17,40]. For comparison, Jurikova et al. [14] found the highest content of chlorogenic acid in the Likernaja (100.9 mg/100 g ww) and Granatnaja (90.62 mg/100 g) varieties. When testing the chlorogenic acid content in pomace samples, the highest values were found for wild *S. aucuparia* and the Bussinka and Sahharnaja varieties, at 4.79 mg/g d.m., 3.64 mg/g d.m. and 3.62 mg/g d.m., respectively. Mikulic-Petkovsek et al. [41] also reported that the Bussinka variety is rich in neochlorogenic acid. In the study by Polka et al. [24], it was noted that hydroxycinnamic acids dominated in *Viburnum opulus* fruits (88.26% of the total phenolic compounds). During their research, Goławska et al. [42] studied different varieties of the fruit of the coral viburnum. Based on the absorption spectra of the chromatograms, eleven phenolic acids were identified—5 hydroxybenzoic acids (gallic acid, *p-*hydroxybenzoic acid, syringic acid, salicylic acid, and benzoic acid) and six hydroxycinnamic acids (chlorogenic acid, caffeic acid, *p-*coumaric acid, ferulic acid, *o*-coumaric acid, and *t-*cinnamic acid). It was shown that *p-*coumaric acid and gallic acid dominated in *V. opulus* plants. The concentration of gallic acid was 1.08–1.12 mg/g d.m. In turn, the concentration of *p-*coumaric acid was 1.83–2.22 mg/g d.m. The content of other hydroxybenzoic and hydroxycinnamic acids was low and similar. During the study, Goławska et al. [42] also studied the flavonoid profile in the fruits of *V. opulus*. They identified nine flavonoids. It was shown that the compounds myricetin (1.60–2.21 mg/g d.m.) and kaempferol (1.54–1.72 mg/g d.m.) were dominant in *V. opulus* plants. Data on the composition of individual phenolic compounds are very important. They show great diversity, which suggests their function. Studies on the qualitative composition of phenolic compounds in *V. opulus* fruits have shown the presence of hydroxybenzoic acids (e.g., gallic acid, vanillic acid, and syringic acid) and hydroxycinnamic acids (e.g., chlorogenic acid, caffeic acid, coumaric acid, and ferulic acid), flavanols (e.g., catechin, epicatechin, and procyanidin), flavonols (e.g., quercetin), and anthocyanins (e.g., cyanidin), and differences in the phenolic composition between the studied genotypes of *V. opulus* fruits have also been demonstrated [43,44]. In their studies, Polka et al. [24] found that, in *V. opulus* fruits, the dominant phenolic compounds are hydroxycinnamic acids (fruits 763.32 mg/100 g d.m.). In the studies, Goławska et al. [42] found that chlorogenic acid is the dominant compound in fruits (752 mg/100 g). Meanwhile, Perovej et al. [43] and Velioglu et al. [45] indicated chlorogenic acids (204 mg/100 g) as the main component of viburnum fruit d.m. and (+)-catechin (29 mg/100 g d.m.). According to the present study and the results of previous studies, *C. monogyna* is also rich in phenolic acids [46,47,48]. In the literature, it was noticed that, among phenolic acids, the dominant ones were chlorogenic acid [49], gallic acid, and caffeic acid [50,51,52,53]. Moreover, *C. monogyna* fruits contained flavonoids such as catechin [48,54,55], quercetin, and kaempferol [56]. A qualitative evaluation of the phenolic compounds’ profile was also carried out in the fruits of Romanian *S. aucuparia* cultivars. According to Aurori et al. [32], the most dominant phenolics were chlorogenic and neochlorogenic acids (704.79 µg/mL and 376.61 µg/mL, respectively). According to previous studies, the main phenolic compounds discovered in the fruits of *S. aucuparia* were represented by these acids [40,57,58,59,60]. Moreover, the other phenolic acids discovered in the fruits of *S. aucuparia* are gallic acid and ferulic acid [33]. Among the flavonoids, Q 3*-O-*rutinoside (rutin) was identified at a concentration of 12.19 µg/mL [32]. This amount is similar to that reported by Rutkowska et al. [60]. Other authors reported higher amounts of this flavonol [59]. Furthermore, Q 3*-O-*glucoside (isoquercetin) was found in lower concentrations than previously reported [61].

The results of the study suggest that these wild fruits are a significant source of polyphenols such as phenolic acids and flavonoids, which may have potential applications in food and pharmaceuticals.

Based on the present study, the content of five organic acids was found in the tested samples (Table 3). The quantitative profile of these acids varied depending on the fruit species. It was noted that citric acid was dominant in the fruits of *C. monogyna*, *S. aucuparia,* and *S. nigra*. While in the fruits of *V. opulus* L., the dominant organic acids were two out of five (malic acid and citric acid). The content of organic acids was also analyzed by other researchers. In their studies, Mikulic-Petkovsek et al. [41] and Tundis et al. [62] noticed small amounts of shikimic, tartaric, and fumaric acids in *S. nigra* fruits. Simple organic acids, primarily malic, citric acid, and oxalic acid, have been reported in the leaves of *S. aria* and *S. aucuparia* [63]. In the literature, it was reported that the fruits of *S. aucuparia* contained citric, malic, tartaric, and fumaric acids [64].

**Figure 3:**
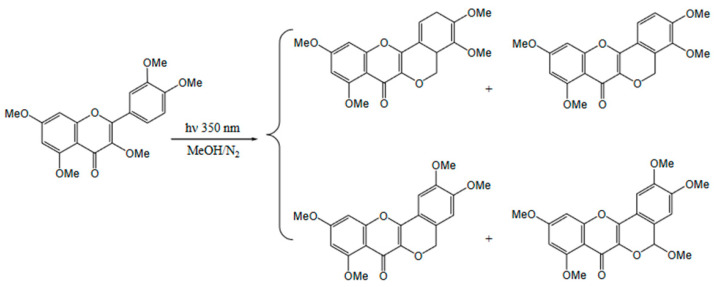
Photochemistry of quercetin [67].

The literature demonstrated the influence of soil location and composition on significant differences in the content of phenolic acids and flavonoid aglycones. The literature indicates these relationships for cultivated plants, not wild plants [65]. Hence, an attempt was made to link the elemental composition of the soil with the level of polyphenols in the fruit. Mineral components do not migrate in the plant but activate the mechanism of the shikimic acid pathway through a stress factor. Interestingly, there is a different concentration of both acid and flavonoid glycosides (Table 4). This fact can be explained by the varied soil composition and increased iron and phosphorus content. This is probably related to the presence of artificial fertilizers in the groundwater. The forest biocenosis accumulates water in periods of excess and releases it to areas located in lower parts of the catchment area in periods of water shortage. The forest limits the runoff of rainwater and transforms it into a slower, underground biological cycle. The forest purifies water; the process takes place in a particularly active environment of forest soils, which act as a biological filter. Another factor influencing the content of glycosides may be the lack of light competition between plants in the habitat from which the fruits were collected. All flavonoids absorb UV waves and accumulate mainly in the epidermis of plant cells, and their biosynthesis into bound forms is usually activated after exposure to UV radiation. In this work, analyses of insolation of any location in Poland were performed using the OnGeo.pl application. The highest insolation was found in the Laski location. At the same time, a significantly higher content of polyphenols in fruits was found at this location. In addition, such forms exhibit strong antioxidant properties at a significantly higher potential than free forms, acting as scavengers of free oxygen radicals produced after the exposure of plant organs to high-fluence UV radiation (high fluence UV exposure). The literature reports indicate that polyphenols can serve as antioxidants other than effective UV suppressors in photoprotection [66]. Flavonoids and phenolic acids in bound form accumulate in response to “excess light” in the presence or absence of UV radiation. Flavanoids substituted with a dihydroxy B ring, namely quercetin 3*-O-* and luteolin 7*-O-*glycosides, accumulate rapidly in response to sunlight intensity in the absence of UV radiation. Changes in the phenylpropanoid concentration in leaves induced by UV radiation affected the antioxidant activity to a greater extent than the UV-shielding capacity of the extracts. Early responses to sudden increases in sunlight include a rapid increase in the concentrations of quercetin derivatives and cyanidin 3*-O-*glucoside, the latter of which absorbs only slightly in the UV region of the spectrum. In contrast, hydroxycinnamates and monohydroxy flavonoids of the B ring do not respond to UV radiation (Figure 3). Flavonoids may therefore have an important antioxidant function in photoprotection. This hypothesis is further supported by the large distribution of quercetin and luteolin derivatives in the mesophyll vacuoles in the corresponding epidermal cell compartments. Future experiments to assess the relative contribution of flavonoids to the complex antioxidant defense systems operating in leaves are needed to help finally resolve the question of the importance of their antioxidant functions in photoprotection.

The bioactive compounds contribute to the antioxidant, antimicrobial, and potentially health-promoting properties of these fruits. The results highlight the importance of understanding the specific bioactive composition of wild fruits for their potential use in nutrition and medicine.

### 2.4. Elemental Composition of Soil

Statistically significant differences (*p* < 0.05) were observed between fruits from different locations in terms of phenol and flavonoid content. Based on the conducted studies, differences were noted in the qualitative and quantitative profile of phenolic compounds between wild plant species. Moreover, quantitative differences were also found within the species, which may be influenced by the location. The soil composition can influence the plants and bioactive compound composition. The elemental composition of the soil is presented in Table 5 below.

## 3. Materials and Methods

### 3.1. Research Material

The research was carried out on 4 species of forest shrub fruits belonging to two families: *Rosaceae Juss*. and *Adoxaceae*. Among the plants of the *Rosaceae Juss* (Table 6), two were selected, i.e., *C. monogyna* and *S. aucuparia*. Among the plants from the *Adoxaceae* family, the following plants were selected: *V. opulus* and *Sambucus nigra*. The fruits were harvested at the Forest Experimental Stations of the Poznan University of Life Sciences (Siemianice, Murowana Goślina, Laski) in September 2023 (Poland, Köppen climate classification: D—continental climate). The fruits were collected in 2 natural locations when fully ripe, in the amount of 0.5 kg from each shrub (a total of 36 samples of ripe fruits were collected). After harvesting, they were frozen and freeze-dried (temperature −53 °C, pressure 0.025 mbar). After drying, the fruits were subjected to chemical analyses, identifying the selected bioactive compounds. The tested material was subjected to the extraction process. The extracts were obtained using an innovative method of double hydrolysis (acidic and alkaline) and extraction of the bioactive compounds that were released from glycosidic bonds with diethyl ether. After evaporation, the dry extracts were dissolved in methanol and used for chromatographic analysis (LC-PDA). Each analysis was performed in triplicate (a total of 108 samples were tested).

### 3.2. Methods

#### 3.2.1. ABTS^+^ Radical-Scavenging Capacity

The spectrophotometric analysis of the ABTS^+^ radical-scavenging capacity was determined according to the method of Re et al. [6]. ABTS^+^ was produced by reacting 2 mM ABTS^+^ (Sigma-Aldrich, St. Louis, MO, USA) in H_2_O with 2.45 mM K_2_S_2_O_8_ (Sigma-Aldrich), and it was stored for 12 h at room temperature in the dark. The ABTS^+^ solution was diluted to give an absorbance of 0.750 ± 0.025 at 734 nm in 0.1 M sodium phosphate buffer (pH 7.4). Then, 1 mL of the ABTS^+^ solution was added to 3 mL of the sample extracts at 100 μg/mL concentrations. The absorbance was recorded for 0.5 h at 734 nm. The extent of decolorization was calculated as a percentage reduction of absorbance.

#### 3.2.2. Extraction of Phenolic Compounds

The samples for the analyses were weighed to 0.20 g. They were placed in sealed 17 mL culture test tubes, where first alkaline and then acid hydrolysis were run. In order to run alkaline hydrolysis, 1 mL distilled water and 4 mL 2 M aqueous sodium hydroxide (Sigma-Aldrich) were added to test tubes. Tightly sealed test tubes were heated in a water bath at 95 °C for 30 min. After cooling (approx. 20 min), the test tubes were neutralized with 2 mL 6 M aqueous hydrochloric acid solution (pH = 2) (Sigma-Aldrich). Next, the samples were cooled in water with ice. The flavonoids were extracted from the inorganic phase using diethyl ether (2 × 2 mL) (Sigma-Aldrich). Formed ether extracts were continuously transferred to 8 mL vials. Next, acid hydrolysis was run. For this purpose, the aqueous phase was supplemented with a 3 mL 6 M aqueous hydrochloric acid solution. Tightly sealed test tubes were heated in a water bath at 95 °C for 30 min. After being cooled in water with ice, the samples were extracted with diethyl ether (2 × 2 mL). The produced ether extracts were continuously transferred to 8 mL vials, after which, they were evaporated to dryness in a stream of nitrogen. Prior to the analyses, the samples were dissolved in 1 mL methanol (Sigma-Aldrich). An analysis was performed using an Aquity H class UPLC system equipped with a Waters Acquity PDA detector (Waters, Milford, MA, USA). Chromatographic separation was performed on an Acquity UPLC^®^ BEH C18 column (100 mm × 2.1 mm, particle size 1.7 μm) (Waters, Drinagh, Ireland). The elution was carried out by gradient using the following mobile phase composition: A: acetonitryl with 0.1% formic acid and B: 1% aqueous formic acid mixture (pH = 2) (Sigma-Aldrich). The concentrations of flavonoids were determined using an internal standard at wavelengths of λ = 320 nm. The concentrations of phenolic acids were determined using an external standard at wavelengths of λ = 280 nm. The compounds were identified based on a comparison of the retention time of the analyzed peak with the retention time of the standard and by adding a specific amount of the standard to the analyzed samples and repeated analysis. The detection limit is 1 μg/g [68].

#### 3.2.3. Chromatographic Analysis

The analysis was performed using an Acquity H class UPLC system equipped with a Waters Acquity PDA detector (Milford, MA, USA). The chromatographic separation was performed on an Acquity UPLC^®^ BEH C18 column (Watersy, Dublin, Ireland) (100 mm × 2.1 mm, particle size 1.7 µm) (Watersy, Dublin, Ireland). The elution was carried out in a gradient using the following mobile phase composition: A: acetonitrile with 0.1% formic acid and B: 1% aqueous formic acid mixture (pH = 2) (Sigma-Aldrich). The concentrations of phenolic compounds were determined with the use of an internal standard at wavelengths λ = 320 nm and 280 nm and finally given in mg/1 g of extract. The compounds were identified by comparing the retention time of the analyzed peak with the standard retention time and by adding a specific amount of standard to the analyzed samples and repeating the analysis. The detection limit was 1 µg/g [69].

#### 3.2.4. Total Phenolic Content (TPC, TAC, TFC)

The total phenolic content was measured with the Folin–Ciocalteu reagent [70]. Two mL (Sigma-Aldrich) of the Folin–Ciocalteau reagent were added to 1 mL of the aqueous extract. After 3 min, the reaction environment was alkalized by adding 10 mL of a 10% sodium carbonate solution (Sigma-Aldrich). After 30 min, the solutions were filled up to 25 mL and their absorbance was measured at a wavelength of λ = 765 nm using a Hitachi U-2900 spectrophotometer (Schaumburg, IL, USA). The results were calculated as the mean of triplicates in mg of phenolic compounds per gram of raw material, expressed as gallic acid equivalent (GAE).

#### 3.2.5. Total Chlorophyll Content

The plant material was thoroughly ground in a mortar containing 3 mL of ethyl alcohol (Sigma-Aldrich), a pinch of sand, and a pinch of CaCO_3_ (Sigma-Aldrich). The solution was quantified into labeled centrifuge tubes. The mortar and pestle were rinsed with another 2 mL of alcohol, which was poured into the same centrifuge tubes. The tubes with the chlorophyll alcohol solution (Sigma-Aldrich) were capped and kept in a dark place until centrifugation. The samples were centrifuged at 9000 rpm for 10 min at room temperature. Next, the supernatant was quantitatively transferred into new labeled centrifuge tubes. Then, 1.9 mL of ethyl alcohol and 0.5 mL of the sample under analysis were poured into spectrophotometer cuvettes. The contents were mixed, and the chlorophyll content was determined with a UV/VIS Excellence 6850 spectrophotometer at wavelengths of 645 nm, 649 nm, 654 nm, and 665 nm. The apparatus was zeroed at the specified wavelength on 2 mL of ethyl alcohol. All of the measurements were triplicated. The formula below was used to calculate the content of chlorophyll a and b:

Chlorophyll (a + b) = [(25.1 × A654) × (V: (1000 × W))] × 4 [mg g^−1^ fresh weight]

where

A645–665—absorbance measured at a wavelength of 649–665 nm;
V—total volume of the extract (mL);
W—weight of the sample (g) [71].

#### 3.2.6. Total Carotenoid Content

Determination of the total carotenoids and β-carotene content was carried out in accordance with Kurasiak-Popowska et al. [72] A mass of 0.25 g of the crushed sample was weighed into a centrifuge tube to which methanol (5 mL) (Sigma-Aldrich) was added. The sample was homogenized using a Polytron homogenizer (Kinematica Polytron PT 3100, Bohemia, NY, USA) at 5000 rpm for 2 min, capped, and centrifuged at 300 g for 10 min to pellet the solids. The methanol extract was then transferred to a separate vial to which 5 mL of hexane/acetone (1:1) (Sigma-Aldrich) was added. The sample was homogenized and centrifuged again, and the hexane/acetone extract was removed and combined with the methanol extract. To the combined extracts were added 10 mL of 30% potassium hydroxide in methanol and heated in a water bath at 60 °C for 1 h. Water was then added. This resulted in 2 immiscible layers. The upper layer containing carotenoids was decanted, dried under a nitrogen stream, and stored at −20 °C until analysis. Before spectrophotometric analysis, the samples were dissolved in methanol/methyl tert-butyl ether (MTBE) (Sigma-Aldrich) (1:1, *v*/*v*) and filtered through a 0.4 μm nylon syringe filter. A quantitative analysis of the total carotenoids was performed using a UV-ViS spectrophotometer (UV5100 Spectro Lab, Poland, Warsaw) at a wavelength of 653 nm. A standard curve was established for beta-carotene. The results are presented in mg/kg.

#### 3.2.7. Total Anthocyanin Content

The total anthocyanin content was measured with the spectrophotometric method described by Giusti and Wrolstad [73]. The results of the measurements were expressed as cyanidin-3-glucoside (C3G). Two g of the product were collected and homogenized for 3 min (20,000 rpm) with 100 cm^3^ of a mixture of methanol and 1.5-molar hydrochloric acid (85:15). The homogenate was centrifuged for 20 min at 4000 rpm. A clear liquid was collected for analysis. Sample dilutions were taken into account during the analyses. The other products were diluted with buffers, so as to make a spectrophotometric measurement within an absorbance range of 0.3–0.8 nm. Depending on the sample, the dilution factor (DF) ranged from 12.5 to 20 nm. All the measurements were triplicated with the UV VIS Excellence 6850 spectrophotometer.

#### 3.2.8. Analyses of Insolation

In this work, analyses of insolation of any location in Poland were performed using the OnGeo.pl application. Thanks to the solar map, it is possible to estimate the amount of solar energy falling on a selected point throughout the year (solar map in the OnGeo.pl Geoportal; https://geoportal-krajowy.pl/ accessed on 14 September 2024).

#### 3.2.9. Statistical Analysis

A statistical analysis was performed using the STATISTICA package (Statistica 13.3 PL 2018, version II). Significant differences were analyzed using the Pearson test, at a significance level of 0.05. The power of discrimination of individual factors was also analyzed using stepwise discriminant analysis. An analysis of the correlation between the analyzed chemical parameters was performed. Ten samples from each location were analyzed in three technical replicates.

## 4. Conclusions

The study focused on determining antioxidant activity, polyphenol content, and other bioactive compounds in the fruits of selected forest shrubs from Poland, including *C. monogyna*, *S. aucuparia*, *V. opulus,* and *S. nigra*. The extraction methods were optimized for these plant materials in order to analyze the secondary metabolites, especially the bioactive compounds with antioxidant properties. The results of the study indicate that the fruits of wild plants are a significant source of polyphenols, which can be used in the food, pharmaceutical, and cosmetics industries and plant protection products. In this study, the content of natural pigments in extracts from the fruits of wild plants was also analyzed, with a particular emphasis on carotenoids, chlorophylls, and anthocyanins, which are responsible for the color of ripe fruits. The study showed significant variability in the content of natural pigments in different species and ripe fruits of wild plants. The quantitative and qualitative profiles of 25 bioactive compounds in the fruits of *C. monogyna*, *S. aucuparia*, *V. opulus,* and *S. nigra* were different. The soil composition was also analyzed, which can affect the composition of plants and bioactive compounds. The finding was that there were different concentrations of phenolic acid glycosides and flavonoids. These differences can be explained by variable soil composition, increased iron and phosphorus content, and competition for light between plants in the environment from which the fruits originated. However, it is worth mentioning that this study should be extended to obtain more detailed conclusions. The fruits of *C. monogyna*, *S. aucuparia*, *V. opulus,* and *S. nigra* are valuable sources of antioxidants, polyphenols, flavonoids, and phenolic acids. The study highlights the variability of the content of bioactive compounds in different species and the genetic differences between plant species.

## Figures and Tables

**Table 1:** Antioxidant activity, the total phenolic content, total phenolic acid content, and total flavonoid content in fruits of wild plants (±SD).

Family of Plants	*Rosaceae Juss.*				*Adoxaceae*			
Plant Species	*Crataegus monogyna* Jacq.		*Sorbus aucuparia* L.		*Viburnum opulus* L.		*Sambucus nigra*	
Location	Siemianice	Murowana Goślina	Siemianice	Murowana Goślina	Laski	Murowana Goślina	Laski	Murowana Goślina
ABTS^+^ (μmol Trolox equivalents/kg d.m. fruit)	2844 ± 54 ^a^	2537 ± 38 ^b^	1244 ± 31 ^c^	1204 ± 28 ^c^	1349 ± 29 ^d^	1257 ± 32 ^c^	1255 ± 39 ^c^	1387 ± 29 ^d^
TPC (mg GAE/100 g d.m. fruit)	4322 ± 43 ^a^	4299 ± 53 ^a^	1855 ± 34 ^b^	1948 ± 42 ^b^	722 ± 37 ^c^	761 ± 33 ^c^	4177 ± 58 ^a^	5042 ± 62 ^d^
TAC (mg CAE/100 g d.m. fruit)	122 ± 19 ^a^	130 ± 17 ^a^	100 ± 14 ^a^	355 ± 17 ^b^	106 ± 16 ^a^	111 ± 13 ^a^	324 ± 23 ^b^	102 ± 23 ^a^
TFC (mg RUTE/100 g d.m. fruit)	1041.6 ± 46.3 ^a^	1057.1 ± 36.4 ^a^	758.3 ± 41.7 ^b^	519.2 ± 33.5 ^c^	148.5 ± 18.3 ^d^	119.3 ± 14.2 ^d^	550.2 ± 26.4 ^c^	765.8 ± 28.3 ^b^

Antioxidant activity (ABTS^+^); the total phenolic content (TPC); total phenolic acid content (TAC); total flavonoid content (TFC); d.m.—dry mass.; SD—standard deviation; a, b, c, d—the same letters in the column mean no significant differences *p* = 0.05.

**Table 2:** The content of natural pigments present in fruits of wild plants (mg/kg of d.m. ± SD).

Family of Plants	*Rosaceae Juss.*				*Adoxaceae*			
Plant Species	*Crataegus monogyna* Jacq.		*Sorbus aucuparia* L.		*Viburnum opulus* L.		*Sambucus nigra*	
Location	Siemianice	Murowana Goślina	Siemianice	Murowana Goślina	Laski	Murowana Goślina	Laski	Murowana Goślina
Carothenoids	211.5 ± 19.2 ^a^	206.3 ± 12.3 ^a^	134.2 ± 18.3 ^b^	151.1 ± 14.6 ^b^	51.8 ± 8.5 ^c^	56.3 ± 5.6 ^c^	142.1 ± 13.6 ^b^	133.7 ± 15.2 ^b^
Chlorophyll	0.65 ± 0.07 ^a^	0.59 ± 0.09 ^a^	0.27 ± 0.08 ^b^	1.24 ± 0.11 ^c^	3.11 ± 0.97 ^d^	1.88 ± 0.09 ^c^	1.66 ± 0.09 ^c^	0.22 ± 0.08 ^b^
Anthocyanins	21.3 ± 4.5 ^a^	20.7 ± 5.6 ^a^	50.8 ± 7.2 ^b^	58.9 ± 8.3 ^b^	8.7 ± 2.5 ^c^	5.6 ± 1.2 ^c^	579.6 ± 53.8 ^d^	517.4 ± 42.6 ^d^

d.m.—dry mass; SD—standard deviation; a, b, c, d—the same letters in the column mean no significant differences *p* = 0.05.

**Table 3:** The content of phenolic compounds in fruits of wild plants (mg/kg d.m. ± SD).

No.	Family of Plants	*Rosaceae Juss.*				*Adoxaceae*			
	Plant Species	*Crataegus monogyna* Jacq.		*Sorbus aucuparia* L.		*Viburnum opulus* L.		*Sambucus nigra*	
	Location	Siemianice	Murowana Goślina	Siemianice	Murowana Goślina	Laski	Murowana Goślina	Laski	Murowana Goślina
1	Gallic acid	<LOD	<LOD	<LOD	<LOD	<LOD	<LOD	8.22 ± 0.87 ^a^	12.01 ± 1.01 ^b^
2	4-hydroxybenzoic acid	<LOD	<LOD	<LOD	<LOD	<LOD	<LOD	7.15 ± 1.00 ^a^	3.94 ± 0.52 ^b^
3	2.5-dihydroksobenzoic acid	<LOD	<LOD	<LOD	<LOD	<LOD	<LOD	<LOD	<LOD
4	Vanilic acid	<LOD	<LOD	<LOD	<LOD	<LOD	<LOD	0.04 ± 0.01 ^a^	0.38 ± 0.02 ^b^
5	Caffeic acid	<LOD	<LOD	<LOD	<LOD	<LOD	<LOD	2.45 ± 0.65 ^a^	6.09 ± 0.07 ^b^
6	Syringic acid	<LOD	<LOD	<LOD	<LOD	<LOD	<LOD	4.37 ± 0,74 ^a^	5.27 ± 0.28 ^b^
7	Coumaric acid	<LOD	<LOD	<LOD	<LOD	<LOD	<LOD	<LOD	<LOD
8	Vanilin	<LOD	<LOD	<LOD	<LOD	<LOD	<LOD	8.16 ± 1.01 ^a^	10.55 ± 1.12 ^b^
9	*p-*Cumaric acid	<LOD	<LOD	<LOD	<LOD	<LOD	<LOD	1.56 ± 0.12 ^a^	1.74 ± 0.12 ^a^
10	Benzoic acid	<LOD	<LOD	<LOD	<LOD	<LOD	<LOD	33.77 ± 3.12 ^a^	16.04 ± 2.11 ^b^
11	Ferulic acid	<LOD	<LOD	<LOD	<LOD	<LOD	<LOD	93.08 ± 5.10 ^a^	65.76 ± 4.01 ^b^
12	Sinapic acid	<LOD	<LOD	<LOD	<LOD	<LOD	<LOD	188.25 ± 8.12 ^a^	197.99 ± 7.45 ^a^
13	*t-*Cinnamic acid	<LOD	<LOD	<LOD	<LOD	1.89 ± 0.21 ^a^	1.55 ± 0.13 ^a^	131.54 ± 7.45 ^b^	135.51 ± 6.38 ^b^
14	Chlorogenic acid	1158.22 ± 15.54 ^a^	1199.7 ± 17.59 ^a^	6.55 ± 0.71 ^b^	8.01 ± 0.61 ^b^	58.22 ± 7.18 ^c^	601.41 ± 14.32 ^c^	405.88 ± 12.05 ^d^	389.84 ± 12.69 ^d^
15	Protocatechuic acid	85.24 ± 84.11 ^a^	88.79 ± 4.98 ^a^	233.5 ± 8.54 ^b^	238.54 ± 6.39 ^b^	50.22 ± 6.25 ^c^	53.47 ± 5.18 ^c^	1.77 ± 0.07 ^d^	2.53 ± 0.04 ^d^
16	Rozmaric acid	<LOD	<LOD	<LOD	<LOD	<LOD	<LOD	1.44 ± 0.01 ^a^	2.17 ± 0.02 ^b^
17	Salicylic acid	<LOD	<LOD	<LOD	<LOD	3.11 ± 0.05 ^a^	2.86 ± 0.05 ^a^	7.03 ± 0.10 ^b^	7.17 ± 0.11 ^b^
18	Apigenin	<LOD	<LOD	<LOD	<LOD	<LOD	<LOD	177.29 ± 10.05 ^a^	188.19 ± 7.98 ^a^
19	Katechin	165.2 ± 4.75 ^a^	170.47 ± 4.95 ^a^	<LOD	<LOD	219.3 ± 8.84 ^b^	195.24 ± 4.85 ^b^	0.49 ± 0.01 ^c^	0.14 ± 0.01 ^c^
20	Kempferol	<LOD	<LOD	<LOD	<LOD	<LOD	<LOD	1.79 ± 0.02 ^a^	3.47 ± 0.02 ^b^
21	Luteolin	<LOD	<LOD	<LOD	<LOD	<LOD	<LOD	36.81 ± 1.38 ^a^	74.4 ± 2.02 ^b^
22	Naringenin	<LOD	<LOD	613.5 ± 7.15 ^a^	610.77 ± 7.26 ^a^	0.23 ± 0.01 ^b^	0.12 ± 0.01 ^b^	77.22 ± 3.29 ^c^	83.23 ± 4.05 ^c^
23	Quercetin	1057.2 ± 10.54 ^a^	1102.74 ± 11.15 ^a^	180.22 ± 7.12 ^b^	182.1 ± 5.54 ^b^	1.97 ± 0.02 ^c^	1.25 ± 0.02 ^c^	819.33 ± 8.13 ^d^	956.38 ± 7.48 ^d^
24	Rutin	588.21 ± 5.56 ^a^	549.33 ± 7.32 ^a^	66.84 ± 3.78 ^b^	68.39 ± 6.48 ^b^	0.88 ± 0.02 ^c^	0.79 ± 0.01 ^c^	1798.22 ± 14.15 ^d^	2044.51 ± 15.65 ^e^
25	Vitexin	287.35 ± 7.24 ^a^	271.4 ± 6.69 ^a^	55.74 ± 5.56 ^b^	58.27 ± 5.65 ^b^	<LOD	<LOD	5.31 ± 0.08 ^c^	2.11 ± 0.04 ^c^
26	Citric acid	322.5 ± 3.28 ^a^	330.77 ± 6.26 ^a^	135.2 ± 3.39 ^b^	133.9 ± 3.45 ^b^	821.18 ± 5.89 ^c^	865.2 ± 6.68 ^c^	3.96 ± 0.12 ^d^	4.16 ± 0.14 ^d^
27	Malic acid	7.59 ± 1.01 ^a^	6.58 ± 0.81 ^a^	120.7 ± 4.52 ^b^	121.8 ± 4.56 ^b^	1340 ± 10.18 ^c^	1502.0 ± 10.65 ^d^	1.01 ± 0.01 ^e^	1.02 ± 0.01 ^e^
28	Quinic acid	20.55 ± 1.02 ^a^	19.36 ± 1.02 ^a^	6.85 ± 0.84 ^b^	6.99 ± 0.89 ^b^	150 ± 7.14 ^c^	138.0 ± 7.58 ^c^	<LOD	<LOD
29	Shikimic acid	1.44 ± 0.15 ^a^	1.28 ± 0.12 ^a^	0.79 ± 0.08 ^b^	0.85 ± 0.02 ^b^	49.0 ± 2.32 ^c^	53.0 ± 3.25 ^c^	0.13 ± 0.03 ^d^	0.33 ± 0.01 ^d^
30	Fumaric acid	6.88 ± 1.01 ^a^	7.02 ± 1.02 ^a^	3.44 ± 0.51 ^b^	3.58 ± 0.32 ^b^	<LOD	<LOD	0.23 ± 0.01 ^c^	0.78 ± 0.02 ^c^

<LOD—<low detection limit; SD—standard deviation; a, b, c, d, e—the same letters in the column mean no significant differences *p* = 0.05.

**Table 4:** Content of flavonoid glycosides. Supercritical CO_2_ extraction (SFE) (mg/kg d.m. ± SD).

No.	Family of Plants	*Rosaceae Juss.*				*Adoxaceae*			
	Plant Species	*Crataegus monogyna* Jacq.		*Sorbus aucuparia* L.		*Viburnum opulus* L.		*Sambucus nigra*	
	Location	Siemianice	Murowana Goślina	Siemianice	Murowana Goślina	Laski	Murowana Goślina	Laski	Murowana Goślina
1	5*-O-*caffeoylquinic acid	0.16 ± 0.01 ^a^	<LOD	1.87 ± 0.02 ^b^	2.33 ± 0.41 ^c^	<LOD	14.71 ± 1.23 ^d^	0.16 ± 0.08 ^a^	0.22 ± 0.02 ^a^
2	quercetin 3*-O-β*-d-galactoside	0.45 ± 0.02 ^a^	12.51 ± 0.51 ^b^	4.22 ± 0.05 ^c^	6.34 ± 0.42 ^c^	11.52 ± 0.75 ^b^	1.28 ± 0.13 ^d^	0.45 ± 0.08 ^a^	0.42 ± 0.02 ^a^
3	quercetin 3-*O*-β-d-glucoside	0.26 ± 0.02 ^a^	7.43 ± 0.56 ^b^	1.87 ± 0.02 ^c^	2.44 ± 0.32 ^d^	6.88 ± 0.65 ^b^	0.23 ± 0.02 ^a^	0.26 ± 0.07 ^a^	0.17 ± 0.02 ^a^
4	1-*O*-feruloylquinic acid	1.33 ± 0.08 ^a^	<LOD	<LOD	<LOD	<LOD	0.07 ± 0.01 ^b^	1.33 ± 0.09 ^a^	1.16 ± 0.03 ^a^
5	caffeoyl-maloyl-quinic acid	0.09 ± 0.01 ^a^	0.41 ± 0.01 ^b^	<LOD	<LOD	0.37 ± 0.06 ^b^	0.11 ± 0.01 ^a^	0.09 ± 0.01 ^a^	0.06 ± 0.01 ^a^
6	1-*O*-caffeoylquinic acid	1.23 ± 0.07 ^a^	<LOD	<LOD	<LOD	<LOD	2.11 ± 0.05 ^b^	1.23 ± 0.06 ^a^	1.11 ± 0.09 ^a^
7	5-caffeoylshikimic acid	0.34 ± 0.07 ^a^	<LOD	<LOD	<LOD	<LOD	1.38 ± 0.05 ^b^	0.34 ± 0.02 ^a^	0.21 ± 0.02 ^a^
8	caffeoyl-maloyl-quinic acid	0.12 ± 0.02 ^a^	0.55 ± 0.02 ^b^	<LOD	<LOD	0.61 ± 0.08 ^b^	2.34 ± 0.06 ^c^	0.12 ± 0.02 ^a^	0.18 ± 0.02 ^a^
9	3-caffeoylshikimic acid	1.31 ± 0.04 ^a^	<LOD	<LOD	<LOD	<LOD	0.31 ± 0.02 ^b^	1.31 ± 0.02 ^a^	1.39 ± 0.03 ^a^
10	3-*O*-feruloylquinic acid	0.24 ± 0.01 ^a^	<LOD	1.98 ± 0.21 ^b^	1.33 ± 0.12 ^c^	<LOD	<LOD	0.24 ± 0.01 ^a^	0.21 ± 0.02 ^a^
11	feruloyl-maloyl-quinic acid	0.08 ± 0.01 ^a^	<LOD	0.33 ± 0.03 ^b^	0.87 ± 0.06 ^c^	<LOD	<LOD	0.08 ± 0.01 ^a^	0.05 ± 0.01 ^a^
12	5-*O*-feruloylquinic acid	0.03 ± 0.01 ^a^	<LOD	3.55 ± 0.12 ^b^	1.11 ± 0.05 ^c^	<LOD	<LOD	0.03 ± 0.01 ^a^	0.06 ± 0.01 ^a^
13	Kaempferol-*O*-dihexoside	1.01 ± 0.02 ^a^	5.51 ± 0.06 ^b^	4.64 ± 0.15 ^b^	3.61 ± 0.51 ^c^	5.81 ± 0.12 ^b^	12.51 ± 0.98 ^d^	1.01 ± 0.02 ^a^	1.16 ± 0.03 ^a^

SD—standard deviation; a, b, c, d—the same letters in the column mean no significant differences *p* = 0.05.

**Table 5:** Elemental composition of soil (mg/kg ± SD).

Location	Chemical Elements										
	P	K	Mg	B	Cu	Zn	Mn	Fe	S	Na	Ca
Siemianice	73.2 ± 2.74 ^a^	73.5 ± 2.95 ^b^	10.1 ± 1.12 ^a^	0.75 ± 0.05 ^a^	1.59 ± 0.07 ^a^	7.12 ± 0.51 ^a^	197 ± 5.75 ^a^	683 ± 5.84 ^b^	12.6 ± 1.12 ^a^	0.42 ± 0.02 ^a^	6.30 ± 0.47 ^a^
Laski	76.2 ± 2.15 ^a^	66.5 ± 1.81 ^a^	13.8 ± 0.59 ^b^	0.71 ± 0.02 ^a^	1.58 ± 0.05 ^a^	7.50 ± 1.05 ^a^	196 ± 4.25 ^a^	715 ± 5.28 ^c^	13.9 ± 1.12 ^b^	0.68 ± 0.02 ^b^	6.59 ± 0.26 ^a^
Murowana Goślina	87.4 ± 2.32 ^b^	78.0 ± 1.18 ^c^	12.5 ± 0.54 ^b^	0.89 ± 0.03 ^b^	1.74 ± 0.04 ^b^	7.26 ± 1.01 ^a^	191 ± 4.35 ^a^	613 ± 5.65 ^a^	12.4 ± 1.02 ^a^	0.71 ± 0.02 ^b^	6.46 ± 2.13 ^a^

SD—Standard deviation; a, b, c—the same letters in the column mean no significant differences *p* = 0.05.

**Table 6:** Research material (photos own).

Family of Plants	Plant Species		Location	Number of Bushes
*Rosaceae Juss.*	*Crataegus monogyna* Jacq.	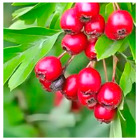	Siemianice 51°05′38″ N 18°11′87″ E	4
			Murowana Goślina 52°34′27.02″ N 17°00′32.14″ E	3
	*Sorbus aucuparia* L.	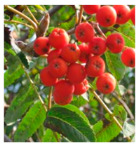	Siemianice 51°05′38″ N 18°11′87″ E	5
			Murowana Goślina 52°34′27.02″ N 17°00′32.14″ E	4
*Adoxaceae*	*Viburnum opulus* L.	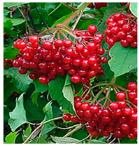	Laski 51°35′26″ N 19°07′58″ E	7
			Murowana Goślina 52°34′27.02″ N 17°00′32.14″ E	3
	*Sambucus nigra*	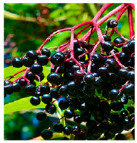	Laski 51°35′26″ N 19°07′58″ E	7
			Murowana Goślina 52°34′27.02″ N 17°00′32.14″ E	3

## Data Availability

Data are contained within the article.
